# Different parental origins of supernumerary X chromosomes in brothers with Klinefelter syndrome: A case report

**DOI:** 10.1097/MD.0000000000017838

**Published:** 2019-11-01

**Authors:** Shin-Hye Kim, Mi-Jung Park, Eun Hae Cho, Sollip Kim, Soo Jin Yoo

**Affiliations:** a Departments of Pediatrics, Sanggye Paik Hospital, Inje University, College of Medicine, Seoul; b Genome Research Center, Green Cross Genome, Yongin; c Departments of Laboratory Medicine, Ilsan Paik Hospital, Inje University College of Medicine, Goyang; d Laboratory Medicine, Sanggye Paik Hospital, Inje University, College of Medicine, Seoul, Republic of Korea.

**Keywords:** 47,XXY, Klinefelter syndrome, Quantitative-fluorescence PCR

## Abstract

**Rationale:**

Recurrence of Klinefelter syndrome (KS) in non-twin brothers is very rare. This study examined the inheritance pattern of supernumerary X chromosomes in non-twin brothers.

**Patient concerns:**

A 16-year-old man presented with small-sized testicles. During his diagnostic work-up, his brother, in his late 20's, also complained of small testes and erectile dysfunction.

**Diagnosis:**

Chromosome analysis in peripheral blood revealed non-mosaic 47,XXY karyotype in both brothers. Their mother showed a normal 46,XX karyotype.

**Interventions:**

To examine the inheritance pattern of supernumerary X chromosomes, quantitative-fluorescence PCR was performed with small tandem repeat markers. It revealed that their supernumerary X chromosomes were inherited from different parents.

**Outcomes:**

After the diagnosis of KS, 2 brothers started to receive testosterone treatment.

**Conclusion:**

This case report is the first to report differences in the origins of supernumerary X chromosomes in brothers with KS and furthers the current understanding of the cytogenetic mechanisms in KS.

## 1 Introduction

Klinefelter syndrome (KS) is the most common sex chromosome aneuploidy affecting 1/500 to 1/1,000 male births. The 47,XXY karyotype in KS sporadically results from nondisjunction of the X chromosome during the first or second meiotic division of gametogenesis in either parent.

Familial KS recurrence is very rare. Most reported familial KS cases involved twin brothers.^[1–3]^ Few studies have reported KS occurrence among non-twin brothers.^[4,5]^ The inheritance pattern of the supernumerary X chromosome in non-twin brothers was investigated in 1 family and their supernumerary X chromosome in brothers were obtained from their father.^[5]^

Here we report the first case of KS in 2 brothers, wherein their supernumerary X chromosomes were inherited from different parents.

## 2 Presenting concerns

A 16-year-old man referred to our pediatric endocrinology clinic because of small-sized testicles. He had a history of right testicular pain accompanying abdominal pain at age of 8 years, and the scrotal ultrasonography finding was normal at that time. Also, he had visited pediatric psychiatric department under the concern of attention deficit at the age of 8 years, but he had never received any long-term behavior therapy or medication. During his diagnostic work-up, his 28-year-old brother who was previously healthy visited the clinic with an erection problem.

## 3 Clinical findings

At presentation, the physical examination of the younger brother revealed a reduced testicular size of 5 cc (right) and 3 cc (left). Development of pubic hair (Tanner 5 stage) and axillary hair (Tanner 3 stage) was appropriate for his age. He was 187 cm tall and weighed 71 kg. A morning serum testosterone level was 4.1 ng/ml, which was within age- and gender-specific reference range (reference range, 1.3–4.3 ng/ml). However, serum luteinizing hormone (LH) and follicle stimulating hormone (FSH) levels were markedly elevated as high as 35.29 mIU/ml (reference range, 1.5–9.0) and 17.62 mIU/ml (reference range, 2.0–9.2), respectively. This patient was the second child born to non-consanguineous, healthy parents. At parturition, his father and mother were 39 and 34 years old, respectively.

His 28-year-old brother also had small testicles (3 cc, both testes) with appropriate pubic hair development on physical examination. He was 178 cm tall. The hormone levels were 23.18 mIU/ml of LH, 41.68 mIU/ml of FSH, 4.2 ng/ml of morning testosterone. At parturition, his father and mother were 28 and 23 years old, respectively.

Both brothers otherwise had normal findings on general physical examination with neither had intellectual nor behavioral issues. His father had died from a lung cancer 3 years ago. The medical histories of the members of this family were otherwise unremarkable; the father and mother were 167 cm and 162 cm tall, respectively.

## 4 Diagnostic focus and assessment

**Figure 1:**
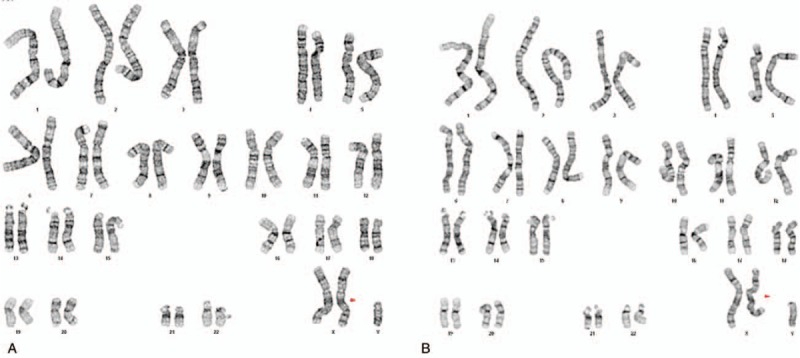
GTG-banded karyotypes of peripheral blood cells of the 2 siblings. (A) The 47,XXY karyotype of the proband; (B) the 47,XXY karyotype of the proband's brother.

Cytogenetic analysis was performed using a phytohemagglutinin-stimulated lymphocyte culture derived from peripheral blood. The proband and his brother presented the 47,XXY karyotype (Fig. 1). Their maternal karyotype was a normal 46,XX karyotype.

**Figure 2:**
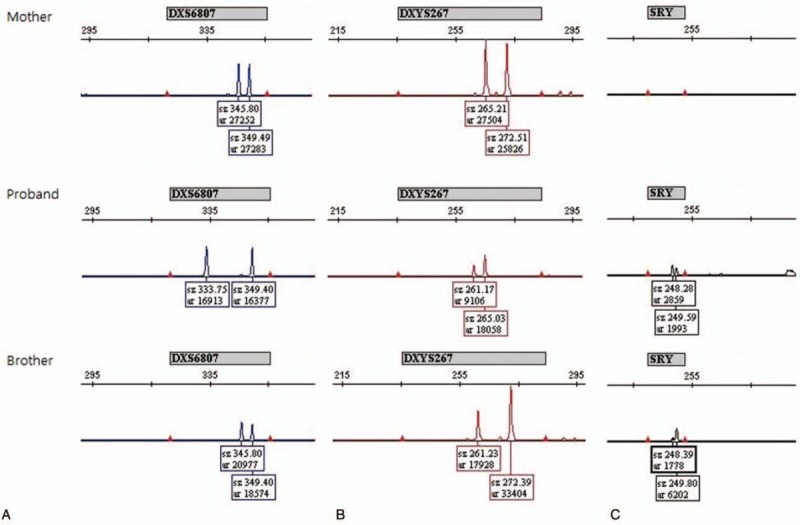
Quantitative fluorescence-PCR analysis of STR markers for X and Y chromosomes and for the *SRY* gene in family members including the proband, his mother, and his brother. Panel (A) shows the result of the DXS6807 marker. The proband has 1 maternal allele. The brother has 2 maternal alleles. Panel (B) shows the result of the DXYS267 marker. Among 2 maternal alleles, the proband has 1 and the brother has another. The proband and his brother share the same allele that is different from the maternal alleles. In panel (C), 2 siblings have 2 identical alleles probably inherited from their father.

To determine the zygosity, quantitative-fluorescence PCR (QF-PCR) with small tandem repeat (STR) markers was performed for the 2 siblings and their mother. DNA was extracted from peripheral bloods using a QIAamp DNA Mini Kit (Qiagene, Hilden, Germany) according to the manufacturer's instructions and 5 ng of DNA was used for PCR. A QF-PCR assay was performed using Elucigene (Gen-Probe, San Diego, CA, USA) for 10 STR markers for chromosomes X and Y including *amelogenin* and *SRY* genes.^[6]^ Amplified samples were analyzed with ABI 3130x1 (Applied Biosystems, Foster City, CA, USA) and Genotyper 3.7 (Applied Biosystems). The STR markers in X and Y chromosomes showed the findings compatible with XXY karyotype (Fig. 2).

Eight markers were read for the X chromosome. In the proband, X chromosome markers were heterozygous and only 1 allele was of maternal origin, suggesting that he received 1 X chromosome from his mother and the other X chromosome from his father. The brother displayed identical patterns with his mother for X chromosomal markers, suggesting maternal nondisjunction of the X chromosomes at meiosis I. Two Y chromosomal markers displayed the same patterns in the 2 siblings suggesting that they were inherited from the same father.

The patients provided written informed consent for the study. This study was approved by the Institutional Review Board of Sanggye Paik Hospital, Inje University.

## 5 Discussion

In KS, the non-mosaic 47,XXY is acquired because of nondisjunction of the sex chromosomes.^[7]^ It occurs slightly more frequently in the mother (56%) during the first or second meiotic division of gametogenesis.^[8]^ The supernumerary X chromosome of paternal origin can occur only during meiosis I. Considering the cytogenetic mechanism, the extra X chromosomes in monozygotic twins presenting non-mosaic KS are presumed to have the same parental origin, i.e., either paternal or maternal.

KS in non-twin brothers has been reported in only 2 families.^[4,5]^ Of these, only the study by Wood et al evaluated the parental origin of the extra X chromosome by haplotype analysis using microsatellites.^[5]^ In that study, the supernumerary X chromosomes in both brothers were inherited from their father.

In our study, the 2 non-twin brothers had the same karyotype of 47, XXY, but the origins of the supernumerary chromosomes were different. The supernumerary X chromosomes in the proband, the younger brother in this family, was presumed to be of paternal origin, suggesting that paternal nondisjunction of the X/Y chromosomes occurred at meiosis I. In his brother, both X chromosomes were derived from his mother, resulting from an error in maternal meiosis I. Two independent nondisjunction events occurred during maternal and paternal meiosis I, respectively, indicating that the recurrence of KS in this family occurred coincidentally through different mechanisms. This is consistent with the current knowledge that most constitutional aneuploidy resulting from nondisjunction occur randomly.

In this family, QF-PCR based on STR markers could elucidate the origins of 2 X chromosomes in the 2 siblings, although paternal specimens were not available. QF-PCR was helpful for investigating the origin of numeric chromosomal abnormalities even if only 1 parent is available.

Meiosis I error occurs more frequently when the supernumerary chromosome is of maternal origin and is associated with advanced maternal age.^[8]^ In this family, maternal age did not affect non-disjunction of the maternal X chromosome. An error in maternal meiosis occurred at the first pregnancy in her twenties, not at the second pregnancy in her thirties.

Wikström et al assessed whether the physical and laboratory findings were different depending on the origin of extra X chromosome.^[9]^ They found that a paternal compared to a maternal origin of the supernumerary X chromosome was associated with a later onset of puberty. Other clinical findings are not significantly different between groups. However, this study result cannot be generalized, because only a small number of KS patients (N, 14) were included. In our case, the proband, the younger brother, had a paternal origin of the supernumerary X chromosome and presented earlier than his sibling with a maternal origin. The elder brother came to know that he was a KS patient in his late 20's only after his younger brother was diagnosed with KS. However, no differences were found in their pubertal onsets upon history taking and their clinical presentations such as the testicular sizes and hormone levels including gonadotropin and testosterone. The phenotypic and clinical effects of the differential parental origin of extra X chromosomes on clinical outcomes in KS requires further investigation in more patients.

In summary, this is the first case report of KS brothers with the supernumerary X chromosomes of different parental origins, which were revealed by QF-PCR analysis with STR markers in the proband, brother, and their mother. We believe that our study provides novel insights into the pathology of the supernumerary X chromosome in KS.

## Author contributions

**Formal analysis:** Mi-Jung Park, Eun Hae Cho.

**Investigation:** Shin-Hye Kim, Eun Hae Cho, Sollip Kim, Soo Jin Yoo.

**Writing – original draft:** Shin-Hye Kim, Soo Jin Yoo.

**Writing – review & editing:** Mi-Jung Park, Sollip Kim, Soo Jin Yoo.

## References

[1] Flannery DB Brown JA Redwine FO Antenatally detected Klinefelter's syndrome in twins. Acta Genet Med Gemellol (Roma) 1984;33:51–6. 6741419 10.1017/s0001566000007492

[2] Hatch TR Moore RJ Klinefelter syndrome in identical twins. Urology 1985;26:396–7. 4049616 10.1016/0090-4295(85)90190-6

[3] Takayasy J Kinoshita K Tsuboi T Twins with Klinefelter's syndrome. Lancet 1967;2:1424. 10.1016/s0140-6736(67)93061-9 4170083

[4] Gustavson KH Gamstorp I Meurling S Bilateral teratoma of testis in two brothers with 47,XXY Klinefelter's syndrome. Clin Genet 1975;8:5–10. 1149322 10.1111/j.1399-0004.1975.tb01947.x

[5] Woods CG Noble J Falconer AR A study of brothers with Klinefelter syndrome. J Med Genet 1997;34:702. 9279771 10.1136/jmg.34.8.702 PMC1051044

[6] Cho EH Park BY Kang YS Validation of QF-PCR in a Korean population. Prenat Diagn 2009;29:213–6. 19165756 10.1002/pd.2190

[7] Harvey J Jacobs PA Hassold T The parental origin of 47,XXY males. Birth Defects Orig Artic Ser 1990;26:289–96. 2090327

[8] Carothers AD Filippi G Klinefelter's syndrome in Sardinia and Scotland. Comparative studies of parental age and other aetiological factors in 47,XXY. Hum Genet 1988;81:71–5. 3198129 10.1007/BF00283733

[9] Wikstrom AM Painter JN Raivio T Genetic features of the X chromosome affect pubertal development and testicular degeneration in adolescent boys with Klinefelter syndrome. Clin Endocrinol (Oxf) 2006;65:92–7. 16817826 10.1111/j.1365-2265.2006.02554.x

